# Enhanced Strength and Sprint Levels, and Changes in Blood Parameters during a Complete Athletics Season in 800 m High-Level Athletes

**DOI:** 10.3389/fphys.2017.00637

**Published:** 2017-08-31

**Authors:** Beatriz Bachero-Mena, Fernando Pareja-Blanco, Juan J. González-Badillo

**Affiliations:** ^1^Faculty of Sport, Pablo de Olavide University Sevilla, Spain; ^2^Physical and Athletic Performance Research Centre, Pablo de Olavide University Sevilla, Spain

**Keywords:** middle-distance, resistance-training, competition, countermovement jump, hematocrit, IGF-1

## Abstract

The purpose of this study was to analyze changes in sprint, strength, hematological, and hormonal parameters in high-level 800 m athletes during a complete athletics season. Thirteen male athletes of national and international level in 800 m (personal best ranging from 1:43 to 1:58 min:ss) participated in this study. A total of 5 tests were conducted during a complete athletics season. Athletes performed sprint tests (20 and 200 m), countermovement jump (CMJ), jump squat (JS), and full squat (SQ) tests. Blood samples (red and white blood profile) and hormones were collected in test 1 (T1), test 3 (T3), and test 5 (T5). A general increase in the performance of the strength and sprint parameters analyzed (CMJ, JS, SQ, 20 m, and 200 m) during the season was observed, with a significant time effect in CMJ (*P* < 0.01), SQ (*P* < 0.01), and 200 m (*P* < 0.05). This improvement was accompanied by a significant enhancement of the 800 m performance from T3 to T5 (*P* < 0.01). Significant changes in some hematological variables: hematocrit (Hct) (*P* < 0.01), mean corpuscular volume (MCV) (*P* < 0.001), mean corpuscular hemoglobin content (MCHC) (*P* < 0.001), white blood cells count (WBC) (*P* < 0.05), neutrophils (*P* < 0.05), monocytes (*P* < 0.05), and mean platelet volume (MPV) (*P* < 0.05) were observed throughout the season. The hormonal response and creatin kinase (CK) did not show significant variations during the season, except for insulin-like growth factor I (IGF-1) (*P* < 0.05). In conclusion, our results suggest the importance of strength levels in middle-distance athletes. On the other hand, variations in some hematological parameters and a depression of the immune system occurred during the season. Therefore, monitoring of the mechanical, hematological and hormonal response in athletes may help coaches and athletes to optimize the regulation of training contents and may be useful to diagnose states of overreaching or overtraining in athletes throughout the season.

## Introduction

Eight hundred-meter running is a middle-distance event where usually both strength and endurance training are performed concurrently. Positive adaptations to concurrent strength and endurance training have been reported in high-level endurance athletes (Paavolainen et al., 1999; Hoff et al., 2002; Mikkola et al., 2007).

Recently, there has been a growing interest in assessing muscle strength in middle and long-distance athletes, due to the demonstrated benefits of resistance training in such athletes (Saunders et al., 2004; Aagaard and Andersen, 2010; Taipale et al., 2010, 2014; Beattie et al., 2014; Ronnestad and Mujika, 2014). For instance, Mikkola et al. (2007) reported that both heavy and explosive resistance training induced improvements in maximal endurance capacity in long-distance runners. Taipale et al. (2010, 2014) showed a positive effect of maximal and explosive strength training performed concurrently with endurance training on the specific performance in recreational endurance runners. Some studies indicate that strength training might positively influence on long-distance running performance by improving running economy (Kelly et al., 2008; Ferrauti et al., 2010). Likewise, plyometric training has been shown to increase muscle tendon stiffness with a coincidental increase in running economy in long-distance runners (Spurrs et al., 2003). Thus, strength training has been proposed as an important complementary training to increase performance in endurance events by improving other factors, such as running economy (Jung, 2003; Legaz-Arrese et al., 2005). On the other hand, vertical jump tests have been widely proposed as an efficient and immediate assessment tool for lower limbs explosive strength (Cormie et al., 2009; Jiménez-Reyes et al., 2014). The countermovement jump (CMJ) test is one of the most common jump tests used because of its validity, reliability and specificity (Markovic et al., 2004; Jiménez-Reyes et al., 2014). Jiménez-Reyes and González-Badillo (2011) analyzed the evolution of jump parameters throughout a complete season in sprint track and field athletes; as a result, they observed that the best performances were obtained in CMJ when the best running performances were achieved during the same period. The same occurred with the worst running competition, which was followed by the worst CMJ performances of the season. Recently, Bachero-Mena et al. (2017) have found significant correlations between running performance in 800 m event and sprint times (20 and 200 m), squat strength and loaded and unloaded jumps height in 800 m high-level athletes. Despite the extensive published data confirming the strong correlation between strength manifestations and endurance performance (Mikkola et al., 2007; Taipale et al., 2010, 2014), there are limited data on the long-term changes in strength variables associated with long-term middle-distance training.

Although, it is well known that the hematological status in athletes is important due to the essential role of blood which facilitates gas transport, buffering and thermoregulation; to our knowledge, there is no available data describing the blood profile among middle-distance athletes during a complete athletics season. The oxygen (O_2_) transport capacity has been found to correlate directly with aerobic performance (Berglund and Hemmingson, 1987). Likewise, a strong correlation between total hemoglobin (Hb) and maximal O_2_ uptake (VO_2max_) has been found in athletes (Sawka et al., 2000; Schmidt and Prommer, 2010). Thus, a high O_2_ transport capacity is a clear advantage for aerobic athletic performance. Some parameters required to evaluate O_2_ transport capacity are: Hb, hematocrit (Hct), and total red blood cell count (RBC) in circulation. Hb and RBC indicate the total amount of O_2_ that can be transported by blood, redirecting O_2_ to organs with a high O_2_ demand while maintaining basal O_2_ supply in less active tissues (Mairbäurl, 2013). An acute reduction of blood Hb concentration, even when the circulating blood volume is maintained, results in lower VO_2max_ and endurance performance, due to the reduction of the O_2_ carrying capacity of blood (Calbet et al., 2006). Conversely, an increase of Hb concentration is associated with enhanced VO_2max_ and endurance capacity that is also proportional to the increase in the O_2_ carrying capacity of blood (Calbet et al., 2006). In this line, Rietjens et al. (2002) evaluated the red blood profile of elite olympic distance triathletes during 3 years to find out that long term endurance training did not largely alter hematological status, but that regular screening of hematological variables was desirable to control the normal range. Despite the well-established relationship existing between packed cell volume, VO_2max_, aerobic performance and maximal exercise capacity (Kanstrup and Ekblom, 1984; Calbet et al., 2006), there is limited data on the long-term changes in hematological variables associated with endurance training. On the other hand, white cells are an important part of the immune system. The immune system may be affected by the level of activity in which an athlete is engaged (Vleck et al., 2014). Through several mechanisms, the immune system may be depressed during intense endurance activity, resulting in an increased risk of illness or infection (Knez et al., 2006).

Strenuous physical exercise promotes a stress-activated response from the endocrine system (Crewther et al., 2006). During long-term training, these responses may be modulated by the physical conditioning that occurs with chronic training (De Souza et al., 1991). Long-term hormonal adaptations to endurance training have been characterized by a decrease, or no change, in the basal concentrations of hormones (Consitt et al., 2002). One of the most studied hormones in response to exercise is testosterone (T), considered as a primary anabolic hormone since it increases protein synthesis and decreases protein degradation (Crewther et al., 2011). Fatigue-induced variations in salivary T have been found in relation to the intensity and duration of a preceding physical load, indicating a catabolic state (Gatti and De Palo, 2011). On the contrary, cortisol (C) is one of the primary catabolic hormones, as it increases protein degradation and decreases protein synthesis (Crewther et al., 2011). In that way, C can be used to determine psychophysiological stress during single and repeated exercise sessions even if a non-univocal relationship has been found between stress and C concentration (Gatti and De Palo, 2011). In relation to middle-distance athletes, Balsalobre-Fernández et al. (2014) found a trend in which the weeks with higher salivary free C concentrations were those in which higher CMJ scores were recorded, suggesting that higher C concentrations (lower than 15.7 ng·ml^−1^) boost CMJ performance. Research has indicated that chronic hormonal adaptations in endurance athletes involve depressed T levels and increased levels of C (Hackney et al., 2003), which indicate a catabolic state in the body that could negatively affect the performance. The reduction in training load and fatigue induces a lower adrenocortical response as well as an improvement of the immunity status, without significant correlations of salivary hormones with training load (Guilhem et al., 2015). On the other hand, previous studies have shown an increase in prolactin (PRL) induced by exercise (Gray et al., 1993; Hickson et al., 1994; Kraemer et al., 1998), which appears to be related to the intensity of exercise (Luger et al., 1992). However, there is controversial literature about the changes of resting and exercise-induced PRL concentration and its association to overtraining states (Rojas-Vega et al., 2011). Growth hormone (GH) and its primary downstream mediator, insulin-like growth factor I (IGF-I), play an important role in formation, maintenance, and regeneration of skeletal muscles (Frystyk, 2010). Positive correlations between circulating IGF-I levels and GH secretion, respectively, and indices of fitness have been observed (Frystyk, 2010). Most studies involving hormonal responses used strength-related athletes. So, to our knowledge, there are no studies monitoring the hormonal response in middle-distance athletes during a complete athletics season.

Therefore, the aim of this study was to analyze the changes in sprint and strength parameters, as well as hematological and hormonal variables during a complete athletics training season in 800 m high-level athletes.

## Methods

### Participants

Thirteen male athletes of national and international level in 800 m (with personal best ranging from 1:43 to 1:58 min:ss) participated in this study (age: 22.9 ± 5.3 years; height: 175.2 ± 5.5 cm; body mass: 62.9 ± 4.4 kg). Two of them were classified 1st and 2nd in the national championship and national ranking; they also had participated in the last Olympic Games. All athletes had completed strength-training programs in the past and were familiarized with the testing procedures. The athletes participated in national and international competitions during the period of testing. Due to the characteristics of the sample (national and international level 800 m athletes), conditioning and training programs were determined entirely by the coaches. No physical limitations or musculoskeletal injuries that could affect testing were reported. This study was carried out in accordance with the recommendations of the Ethics Committee of Pablo de Olavide University, Seville, Spain. All participants were fully informed about procedures, potential risks and benefits of the study and they all signed written informed consent in accordance with the Declaration of Helsinki prior to the tests.

### Experimental design

**Figure 1:**
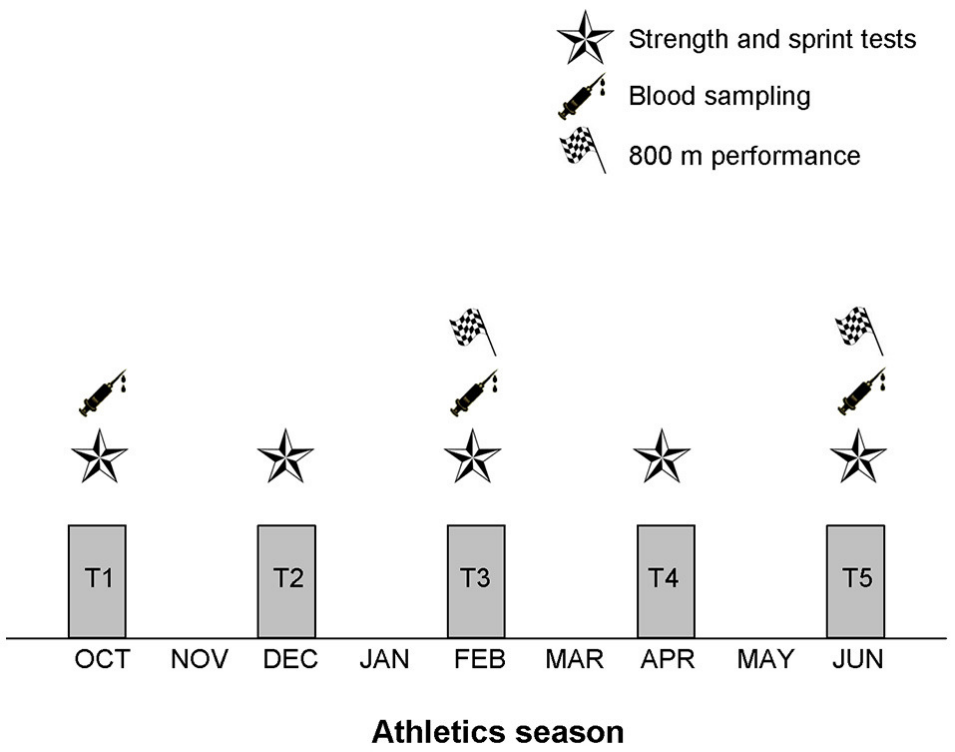
Overview of the experimental design. A total of five tests were spread over the athletics season. Strength and sprint tests were performed in all the tests. Blood samples were collected in T1, T3, and T5. Eight-hundred meter performance was recorded in T3 and T5.

During a complete athletics season, strength and sprint tests were carried out every 2 months (a total of 5 tests were performed) from October to June (Figure 1). Likewise, blood samples for determining basal hematological and hormonal profile were collected in T1, T3, and T5, completing a total of 3 samples during the whole athletics season. The testing was performed in two sessions separated by 1 week. Session 1 consisted of blood sampling, sprint test (20 m) and strength tests: countermovement jump (CMJ), jump squat (JS), and full squat (SQ). Session 2 consisted of 200 m test. Testing sessions were always carried out after a full day of rest, at the same time of the day.

### Procedures

#### Test preparation

During the first testing day, all the participants completed a 20 min standardized warm-up protocol consisting of 10 min of low intensity jogging, 5 min of joint mobilization exercises, and one 40 m sprint at 80% effort, two 20 m sprint at 90% effort, and one 10 m sprint at 100% effort with 2 min rest between them. After the sprint test, the athletes performed the strength tests in the following order: CMJ, JS, and SQ; in addition to the standardized warm-up, the participants did SQ without external load and 5 progressive CMJ. On the second testing day, the athletes performed the 200 m.

#### Sprint testing

In the first day two 20 m trials were performed. The sprint trials were recorded by photocells (Racetime 2; Microgate, Bolzano, Italy), based on a radio impulse transmission system and a reflection system. Runs were performed from static biped start position with the start line located 1 m behind the first photocell. The rest of the photocells were placed at 10 and 20 m. The best time of the two 20 m trials was recorded. The rest period between sets lasted 3 min. The sprint test (20 m) was conducted on a synthetic running track in an indoor hall.

#### Jump measurements (CMJ and JS)

The CMJ and JS were performed on an infrared platform Optojump (Microgate, Bolzano, Italy) that calculated jump height *(h)* through flight time (*t*) and the acceleration due to gravity (*g*) as follows: *h* = *t*^2^ × *g/8*. The CMJ was performed with both hands on the waist, while making a downward movement approximately to 90°-knee flexion followed by a vertical jump of maximum effort. The participants were required to do 3 trials separated by 1 min rest, mean height being recorded. Just after the CMJ test, the JS test was performed with progressive loads ranging from 20 kg up to the load allowing the participant to jump up no more than 20 cm high. The JS test was performed using a Smith machine (Multipower Fitness Line, Peroga, Murcia, Spain), which allows a smooth vertical displacement of the bar along a fixed pathway. The athletes performed two JS separated by 2 min rest with each load. The mean heights of the 2 jumps of each of the common loads performed in the 5 tests were used for the subsequent statistical analysis.

#### Full squat test (SQ)

After the JS test, an incremental loading full SQ test was performed on a Smith machine (Multipower Fitness Line, Peroga, Murcia, Spain) using a linear velocity transducer (T-Force System; Ergotech, Murcia, Spain). Instantaneous velocity was sampled at a frequency of 1,000 Hz. After a warm-up, initial load was set at 20 kg for all participants and was gradually increased in 10 or 5 kg increments until the attained mean propulsive velocity was ~1 m·s^−1^ (55–60% 1RM), which was considered a sufficient load to evaluate lower limb strength (Conceição et al., 2015). This resulted in a total of 4.1 ± 0.7 increasing loads performed by each athlete. The participants started from the upright position with their knees and hips fully extended, the barbell resting across their back at the level of the acromion. Each participant descended in a continuous motion until the top of the thighs got below the horizontal plane, and then they immediately reversed the motion and ascended back to the upright position. The participants were always required to execute the concentric phase of SQ in an explosive manner, at maximal intended velocity. For each load, the repetition correctly performed at the highest velocity was recorded. The velocities reported in this study correspond to the average velocity of the propulsive phase (Sánchez-Medina et al., 2010) for each common load performed in the 5 tests, from 20 kg to the load of ~1 m·s^−1^.

#### Two hundred meter running test

This test was performed on a synthetic running outdoor track (Mondo). Wind conditions were monitored constantly by an Oregon Scientific WMR-918 (Oregon Scientific, Tigard, OR, USA) meteorological station. A mathematical model (Quinn, 2003) was used in order to adjust the potential influence of wind in the time performances. This mathematical model suggests that a head wind of −2.0 or −1.0 m·s^−1^ causes a time lost of 0.121 and 0.059 s respectively, and that a tail wind of +2.0 or +1.0 causes a time gain of 0.112 and 0.056 s respectively. Just one 200 m trial was performed. The procedures were the same as the above mentioned for the 20 m test. Two pairs of photocells (Racetime 2; Microgate, Bolzano, Italy) were used for timing recordings.

#### Eight hundred meter performance

The best 800 m time performance obtained by the athletes in the nearest competition (within 2 weeks) to the tests T3 (winter competition season) and T5 (summer competition season) was recorded for the analysis.

#### Blood sampling

Blood samples were collected during T1, T3, and T5. In view of large fluctuations that steroids concentration may exhibit (Gatti and De Palo, 2011), blood samples were always collected at the same time in the afternoon, when hormones concentrations exhibit reduced fluctuations in comparison with morning measurements. The participants were asked to rest from training the day before the sampling and the tests and they were asked to consume the same foods and fluids at the same time from 12 h prior to each subsequent test day. Before blood sampling the athletes rested on a bed for at least 10 min. Blood samples were taken from the athlete in supine position from the antecubital vein by a qualified laboratory technician using the Vacuette system and collected in tubes containing EDTA K 3 (3 ml) or no anticoagulant (5 ml), for hematological and hormonal analysis, respectively. Hematological analysis was performed using an XN-9000 analyzer (Roche Diagnostics, Indianapolis, USA). The following hematological variables were determined: hemoglobin (Hb), hematocrit (Hct), red blood cell count (RBC), mean corpuscular hemoglobin (MCH), mean corpuscular hemoglobin content (MCHC), mean corpuscular volume (MCV), red blood cell distribution width (RDW), white blood cells count (WBC), neutrophils, lymphocytes, monocytes, eosinophils, basophils, platelets, and mean platelet volume (MPV). Concentrations of total testosterone (T), cortisol (C), insulin-like growth factor I (IGF-1), human growth hormone (GH), prolactin (PRL), and creatin kinase (CK) were measured using electrochemiluminescence immunoassays on the Cobas E170 autoanalyzer (Roche Diagnostics, Indianapolis, USA).

### Statistical analyses

All the data are reported as mean value ± standard deviation (SD). Test-retest absolute reliability was measured by the coefficient of variation (CV), whereas relative reliability was assessed by the intraclass correlation coefficient (ICC) using the one-way random effects model, and confidence interval (CI) at 95%. ICC values ≥0.9 were interpreted as very reliable (Vincent, 1999). A CV of ≤10% was set at the criterion to declare a variable as reliable (Augustsson et al., 2006). Reliability analyses were done from T1 data. The normal distribution of the data was verified with the Shapiro-Wilk test. Statistical analyses for changes throughout the season were assessed using one-way repeated measures analysis of variance (ANOVA). The main effects were compared using the *post hoc* Bonferroni method. Statistical significance was set at *P* ≤ 0.05. SPSS for Mac (IBM Corporation, New York, NY, USA) (release 20.0.0) was used for all statistical analyses.

## Results

Mean and SD data of the different parameters (sprint and strength, hematological, and hormonal) measured during the 5 tests throughout the complete athletics season are presented in Tables 1–3, respectively.

**Table 1:** Changes in strength and running variables during a complete athletics season in 800 m high-level athletes.

**Strength and running variables**	**T1**	**T2**	**T3**	**T4**	**T5**
CMJ (cm)	40.0 ± 6.2^##^	40.0 ± 6.6	40.9 ± 7.4	41.9 ± 5.9	43.0 ± 6.4^aa^^,^^bb^^,^^c^
JS (cm)	21.3 ± 2.7	21.9 ± 2.9	22.6 ± 3.8	22.9 ± 3.4	23.0 ± 4.2
SQ (m·*s*^−1^)	1.19 ± 0.07^##^	1.23 ± 0.09	1.26 ± 0.10	1.32 ± 0.05^aaa^^,^^b^	1.28 ± 0.05^a^
20 m (s)	2.93 ± 0.10	2.92 ± 0.11	2.92 ± 0.10	2.91 ± 0.08	2.88 ± 0.06
200 m (s)	–	24.12 ± 1.13^#^	23.89 ± 1.36	23.48 ± 0.86^bb^	23.26 ± 1.03^bb^
800 m (s)	–	–	115.50 ± 4.77^##^	–	113.79 ± 4.14^cc^

a P < 0.05;

aa P < 0.01;

aaa P < 0.001 (significant differences respect to T1);

b P < 0.05;

bb P < 0.01; (significant differences respect to T2);

c P < 0.05;

cc P < 0.01; (significant differences respect to T3). Time effect:

# P < 0.05;

## *P < 0.01. CMJ, countermovement jump; JS, jump squat; SQ, average velocity attained with common loads in full squat test; 20 m, time achieved in 20 m sprint; 200 m, time achieved in 200 m sprint; 800 m, time achieved in 800 m running*.

Reliability was set with ICC and CV for the 20 m sprint and jump tests. Sprint in 20 m was very reliable (ICC: 0.97; CI: 0.92–0.99; CV: 0.7%). Both CMJ (ICC: 0.99; CI: 0.99–1.00; CV: 1.8%) and JS (ICC: 0.97; CI: 0.90–0.99; CV: 5.2%) showed good reliability as well.

### Strength and sprint variables

Concerning the strength variables, significant increases in CMJ from T1 to T5 (*P* < 0.01), from T2 to T5 (*P* < 0.01), and from T3 to T5 (*P* < 0.05) have been found, indicating a tendency to increase throughout the season. As regards the 200 m, a significant decrease in time from T2 to T4 (*P* < 0.01) and to T5 (*P* < 0.01) was observed (during T1 200 m was not performed). The tendency is a decreasing time in 200 m as the season goes on. In the SQ, the average velocity tended to increase throughout the tests, with significant differences from T1 to T4 (*P* < 0.001), from T2 to T4 (*P* < 0.05), and from T1 to T5 (*P* < 0.05). No significant differences were found in 20 m and JS during the season, however, we can observe a progressive tendency to a decrease of the 20 m sprint time throughout the season (T1: 2.93; T2: 2.92; T3: 2.92; T4: 2.91; T5: 2.88 s), and to an increase of the performance in the JS with common loads (T1: 21.3 T2: 21.9; T3: 22.6; T4: 22.9; T5: 23.0 cm) (Table 1).

### Eight hundred meter performance

A significant decrease in 800 m time from T3 (winter competition season) to T5 (summer competition season) can be observed (*P* < 0.01) (Table 1).

### Hematological variables

Concerning the hematological variables, a significant decrease occurred in Hct (*P* < 0.01) from T3 (44.9%) to T5 (42.6%). Likewise, MCV decreased significantly from T1 to T5 (*P* < 0.001), and from T3 to T5 (*P* < 0.001). A significant increase in MCHC from T1 to T5 (*P* < 0.001) and from T3 to T5 (*P* < 0.001) can be observed. RBC, Hb, MCH, and RDW remained statistically unaltered. Significant decreases can be observed from T3 to T5 in WBC, neutrophils, and monocytes (*P* < 0.05). Significant increases in MPV occurred from T1 to T5 (*P* < 0.05) and from T3 to T5 (*P* < 0.05). No significant differences between tests have been found in lymphocytes, eosinophils, basophils, and platelets (Table 2).

**Table 2:** Changes in hematological parameters during a complete athletics season in 800 m high-level athletes.

**Hematological variables**	**T1**	**T2**	**T3**	**T4**	**T5**
RBC (mill·mm^3^)	5.05 ± 0.41		5.14 ± 0.29		5.09 ± 0.38
Hb (g·dl^−1^)	15.11 ± 0.75		15.32 ± 0.64		15.46 ± 0.88
Hct (%)	43.83 ± 2.32^##^		44.86 ± 1.51		42.64 ± 2.11^cc^
MCV (fl)	87.04 ± 4.73^###^		87.52 ± 4.21		84.02 ± 3.64^aaa^^,^^ccc^
MCH (pg)	30.10 ± 1.71		30.22 ± 1.16		30.50 ± 1.78
MCHC (%)	34.49 ± 1.18^###^		34.17 ± 1.36		36.26 ± 1.08^aaa^^,^^ccc^
RDW (%)	12.72 ± 0.53		12.77 ± 0.41		12.81 ± 0.50
WBC (mil·uL^−1^)	6.78 ± 1.92^#^		6.83 ± 2.24		5.92 ± 1.65^c^
Neutrophils (mil·uL^−1^)	3.51 ± 1.42^#^		3.58 ± 1.45		2.91 ± 0.92^c^
Lymphocytes (mil·uL^−1^)	2.48 ± 1.03		2.46 ± 0.83		2.32 ± 0.73
Monocytes (mil·uL^−1^)	0.59 ± 0.18^#^		0.61 ± 0.18		0.52 ± 0.13^c^
Eosinophils (mil·uL^−1^)	0.16 ± 0.08		0.14 ± 0.07		0.14 ± 0.07
Basophils (mil·uL^−1^)	0.03 ± 0.05		0.03 ± 0.05		0.05 ± 0.05
Platelets (mil·mm^3^)	224.38 ± 56.62		225.15 ± 57.00		209.77 ± 56.83
MPV (fl)	11.34 ± 1.03^#^		11.34 ± 1.01		11.68 ± 1.15^a^^,^^c^

a P < 0.05;

aaa P < 0.001 (respect to T1);

c P < 0.05;

cc P < 0.01;

ccc P < 0.001 (respect to T3). Time effect:

# P < 0.05;

## P < 0.01;

### *P < 0.001. RBC, red blood cell count; Hb, hemoglobin; Hct, hematocrit; MCV, mean corpuscular volume; MCH, mean corpuscular hemoglobin; MCHC, mean corpuscular hemoglobin content; RDW, red blood cell distribution width; WBC, white blood cells count; MPV, mean platelet volume*.

### Hormonal and biochemical response

IGF-1 showed a significant decrease throughout the season (*P* = 0.02). Besides, C showed variations near to significance during the season (*P* = 0.09). No significant differences were found in the rest of the hormonal variables analyzed and CK during the season (Table 3).

**Table 3:** Changes in biochemical and hormonal response during a complete athletics season in 800 m high-level athletes.

**Biochemical and hormonal response**	**T1**	**T2**	**T3**	**T4**	**T5**
CK (U·L^−1^)	234.1 ± 142.6		257.9 ± 101.6		202.0 ± 111.5
PRL (ng·ml^−1^)	11.1 ± 6.0		12.3 ± 6.0		12.5 ± 4.3
GH (μg·L^−1^)	0.75 ± 0.94		0.74 ± 1.66		0.56 ± 1.31
IGF-1 (nmol·L^−1^)	37.7 ± 12.5^#^		36.4 ± 12.7		33.8 ± 3.2^a^
C (nmol·L^−1^)	367.8 ± 98.5		322.8 ± 77.8		347.9 ± 88.6
T (nmol·L^−1^)	19.4 ± 4.4		18.3 ± 4.8		20.6 ± 6.7

a P < 0.05 (respect to T1). Time effect:

# *P < 0.05. CK, creatin kinase; PRL, prolactin; GH, human growth hormone; IGF-1, insulin-like growth factor I; C, cortisol; T, testosterone*.

## Discussion

The main purpose of this study was to analyze changes in sprint, strength, hematological, and hormonal parameters during a complete athletics season in 800 m high-level athletes. On the one hand, we observed an increase in strength and sprint performance during the season. This improvement was accompanied by a significant enhancement of the 800 m performance from T3 to T5. Likewise, significant changes in some hematological variables (Hct, MCV, MCHC, WBC, neutrophils, monocytes, and MPV) were detected. On the other hand, the hormonal response and CK did not show significant variations during the season, except for IGF-1. To our knowledge, this is the first study to present the mechanical, hematological and hormonal response during a complete athletics season in elite middle-distance athletes.

The current data indicate that an improvement in lower limb strength could benefit performance in middle and long-distance athletes (Saunders et al., 2004; Aagaard and Andersen, 2010; Taipale et al., 2010, 2014; Beattie et al., 2014; Ronnestad and Mujika, 2014). Our findings support this hypothesis since significant increases in CMJ, SQ, and 200 m performance occurred throughout the season, together with an improvement in 800 m performance. In this line, Jiménez-Reyes and González-Badillo (2011) found out that the best performances in sprinters from 100, 200, and 400 m events were achieved during the same period as when the best performances in CMJ, JS, and SQ were obtained. The same occurred with the worst running competition, which was followed by the worst CMJ, JS, and SQ performances of the season. However, these events are shorter than the 800 m. In accordance with our findings, Hudgins et al. (2013) observed significant correlations between jumping ability and 800 m race time in competitive runners. Likewise, significant correlations between CMJ, JS, and SQ with performance in 800 m in high-level athletes have been found (Bachero-Mena et al., 2017). Contrary to our study, Balsalobre-Fernández et al. (2015) did not find significant improvements in strength parameters during an athletics season in middle and long-distance athletes. Those significant changes in force production in the athletes in our study may be explained by the fact that most of the athletes followed strength-training programs in addition to the typical specific endurance training sessions throughout the season. These strength-training programs consisted mostly in moderate load and low number of repetitions, each repetition always being performed at maximal velocity. However, in Balsalobre-Fernández et al. (2015) athletes conducted a resistance-training program based on strength-endurance with multiple exercises and high repetitions per set (up to 20 RM). The differences observed between the resistance-training programs and the differences in the sample (800 vs. 1,500 m athletes) could explain the discrepancies reported in the evolution of the mechanical parameters throughout the season in both studies. Regarding the sprint variables, a significant improvement of performance in 200 m throughout the season can be observed. This result shows that the distance of 200 m is probably more related to the performance in 800 m than other shorter distances as 20 m. Similarly, significant correlations between 800 and 300 m running times (Deason et al., 1991), and between 800 and 200 m have been found (Bachero-Mena et al., 2017). Therefore, strength and sprint-related variables seem to be relevant in the 800 m performance.

Concerning the hematological variables, a significant decrease in Hct occurred during the summer competition season (from T3 to T5). Rietjens et al. (2002) observed that Hct values tended to be highest during the training season compared to the competitive season in elite olympic distance triathletes. Those results could be explained by the quite large seasonal variations in Hct (relative change up to 15%), since the values observed in summer were lower than those observed in winter, which might result in seasonal changes from ≈48% in winter to ≈42% in summer (Mairbäurl, 2013). Studies of seasonal changes in Hct of athletes are sparse but indicate that Hct might be decreased by another 1–2% in summer by the addition of a training effect (Mairbäurl, 2013). In addition, the decrease in Hct during the summer season could be explained by an expansion of the plasma volume due to the repeated heat exposure, without influence on erythrocyte volume (Sawka et al., 2000), since the athletes training sessions were performed in a city with a range of temperatures from 15–20° in winter to 30–35° in summer. Together with Hct, MCV decreased significantly throughout the season, probably also influenced by hemodilution. Additionally, MCHC increased significantly throughout the season. MCHC is a related variable to Hb and Hct, being a measure of the concentration of hemoglobin in a given volume of packed red blood cell. In that sense, an increase in MCHC would be explained by an increase in Hb and a decrease in Hct, leading to a better O_2_ transport capacity, which might improve performance. No significant differences between tests were found in the other blood parameters related to O_2_ transport (RBC, Hb, MCH, and RDW). In this regard, Rietjens et al. (2002) found a significant reduction of mean RBC from the preparatory to the competitive season. This observed reduction in RBC is also in line with other studies in elite runners, cyclists, swimmers and triathletes (O'Toole et al., 1988; Guglielmini et al., 1989; Schumacher et al., 2000). Hb is one of the most studied physiological variables in relation to induced-endurance training adaptations. In our study, non-significant changes occurred in Hb (*P* = 0.2). Similarly, studies on long term (4–12 months) training showed no or only small effects in Hb (Ray et al., 1990; Green et al., 1991); however, a longitudinal study on “leisure sportsmen” revealed an increase in Hb by 6% during a 9-month endurance training (Schmidt and Prommer, 2008), suggesting that changes of Hb and RBC by training are slow, and that several years of training may be required for a pronounced increase. The fact that our sample consisted of high-level 800 m athletes with high baseline values of Hb (15.11 ± 0.75 g·dl^−1^) could explain the only small changes of this variable during the season. On the other hand, significant decreases of Hb, Hct, and RBC have been observed in male cyclists when increasing the training load (Schumacher et al., 2000). In regard to white cells, while acute exercise bouts have been implicated in an augmented inflammatory state (Kakanis et al., 2010), high levels of physical activity have been linked to reduced systemic inflammation, and aerobic exercise training has been shown to decrease WBC counts (Michishita et al., 2010) and it is associated to inflammatory biomarkers (De Gonzalo-Calvo et al., 2012). In our study significant decreases occurred in WBC, Neutrophils, and Monocytes from T3 to T5, suggesting a depression of the immune system at the end of the season. This finding is in line with Horn et al. (2010), who observed a decreased number of Monocytes and WBC in triathletes. This depressed response observed in our study could be due to the more intense and specialized exercises performed during the summer competition period, and could increase the risk of illness or infection. Besides, the significant increase of MPV throughout the season is consistent with the well-established evidence that aerobic physical activity is effective to enhance circulating activated platelets (Knudsen et al., 1982; Hilberg et al., 2008; Whittaker et al., 2013).

As regards the hormonal and biochemical response, IGF-1 showed a significant decrease throughout the season. Studies of the chronic effects of exercise on the circulating IGF-I have yielded inconsistent results. Some cross-sectional studies reported positive associations between VO_2max_ and immunoreactive IGF-I levels (Poehlman and Copeland, 1990; Poehlman et al., 1994; Eliakim et al., 1996), suggesting that an improvement in fitness would result in a higher serum IGF-I (Frystyk, 2010). By contrast, other studies have shown reductions in immunoreactive IGF-I levels after several weeks of exercise despite an improved physical performance (muscle strength and/or VO_2max_) (Eliakim et al., 1996, 1998). A possible reason for the decrease in IGF-1 could be the state of fatigue induced by the more intense and specialized exercises performed during the summer competition period. The rest of the hormonal parameters did not show significant variations. PRL and GH share similar sequence homology and immune system activities (Gala, 1991), and therefore may be important factors involved in the recovery from exercise-induced muscle disruption. In this line, Smallridge et al. (1985) found that plasma PRL response after thyrotropin-releasing hormone challenge was augmented in endurance-trained male joggers and marathoners compared to male sedentary controls. On the other hand, C and T are hormonal parameters used to determine psychophysiological stress during exercise. In our study, C and T did not show significant variations in spite of the changes of training load during the different training periods in the season. Similarly to our results, other authors did not find significant changes in C during the season in track and field athletes (Guilhem et al., 2015). Nevertheless, in our study C showed a near to significance increase at the end of the season (*P* = 0.09). Balsalobre-Fernández et al. (2015) showed as well a significant increase in C at the end of the season during the competition period. Guilhem et al. (2015) analyzed salivary hormones in track and field athletes of different disciplines (sprinters, jumpers, and middle distance athletes) during a 4.5 months period including a preparation period of 3 months and a pre-competitive period of 1.5 month. They found significant increase of salivary T at week 5 in comparison to week 2, week 3, and week 4 of the pre-competitive period, which could be explained by the load diminution during the competition season. Similarly, Taipale et al. (2014) found a significant increase in T in recreational endurance runners between weeks 0 and 12 after mixed maximal and explosive training, followed by a significant return to baseline between weeks 12 and 16; however C remained statistically unaltered. Contrary to our study, in the studies referred to above, T and C were obtained by salivary markers, which could explain some differences in the results. Regarding the muscle damage markers (CK), no significant changes were found during the season. In Guilhem et al. (2015) significant increase in CK activity occurred from the preparation to the pre-competitive period, which could be due to higher intensity and specialized exercises during this period, which could have increased the exercise-induced muscle damage (Fiorentino et al., 2014). Nonetheless, CK is recognized for being influenced by factors other than the level of muscle damage (e.g., soft tissue trauma, exercise-induced hemoconcentration and/or hemodilution, alterations of tissue clearance; Bleakley et al., 2012).

In conclusion, a complex induced-training response was observed during the complete athletics season in high-level middle-distance athletes, indicated by changes in the mechanical, hematological and hormonal parameters measured. There was a general increase in strength and sprint parameters analyzed (CMJ, JS, SQ, 20, and 200 m) during the season. This improvement in the strength and sprint parameters was accompanied by a significant enhancement in the 800 m performance. Moreover, significant variations in some hematological parameters analyzed (Hct, MCV, MCHC) and a depression of the immune system (WBC, Neutrophils, Monocytes, and MPV) were observed during the athletics season. On the other hand, the hormonal response and CK remained unaltered during the season, except for IGF-1. These interesting results may indicate that the neuromuscular adaptations responsible for the improvement in lower limb strength and sprint could have partially influenced the enhancement of the 800 m performance, which reflects the importance of strength levels in middle-distance athletes. Important physiological adaptations also occurred during the complete athletics season in 800 m high-level athletes. Our findings suggest that the monitoring of the mechanical, hematological and hormonal response in athletes may help coaches and athletes to optimize the regulation of training contents throughout the season and may be useful to diagnose states of overreaching or overtraining in athletes. However, as a limitation of the study, the conclusions concerning blood sampling should be taken cautiously, since only three time points distributed over 8 months were analyzed. Thus, future studies should perform more tests to analyze more exhaustively the effects of the training loads on physical performance and physiological adaptations in order to optimize training contents and prevent non-desirable physical states that could affect performance in high-level athletes.

## Author contributions

Substantial contributions to the conception or design of the work: BB and JG. Acquisition, analysis, or interpretation of data for the work: BB, FP, and JG. Drafting the work or revising it critically for important intellectual content: BB, FP, and JG. Final approval of the version to be published: BB, FP, and JG. Agreement to be accountable for all aspects of the work in ensuring that questions related to the accuracy or integrity of any part of the work are appropriately investigated and resolved: BB, FP, and JG.

### Conflict of interest statement

The authors declare that the research was conducted in the absence of any commercial or financial relationships that could be construed as a potential conflict of interest.
